# Distinguishing Sichuan Walnut Cultivars and Examining Their Relationships with *Juglans regia* and *J. sigillata* by FISH, Early-Fruiting Gene Analysis, and SSR Analysis

**DOI:** 10.3389/fpls.2020.00027

**Published:** 2020-02-25

**Authors:** Xiaomei Luo, Jingyuan Chen

**Affiliations:** College of Forestry, Sichuan Agricultural University, Chengdu City, China

**Keywords:** microsatellite marker, karyotype analysis, natural hybrids, artificial hybrids, walnuts

## Abstract

Walnuts are economically important tree species in Sichuan Province (China) that provide heathy nuts. Fluorescence *in situ* hybridization (FISH) and analyses of an early-fruiting gene fragment and simple sequence repeats (SSRs) were used to distinguish Sichuan walnut cultivars and examine their relationships with *Juglans regia* L. and *Juglans sigillata* Dode. Thirty-four small chromosomes were counted in four Sichuan walnut cultivars. In the four cultivars, 5S rDNA was located in the proximal regions of two chromosomes (5 and 6), while (AG_3_T_3_)_3_ was located at both ends of each chromosome. The existence of the signal at both chromosome ends ensured accurate chromosome counts. 5S rDNA and (AG_3_T_3_)_3_ were not effective in identifying Sichuan walnut cultivars. Evolutionary analysis involving 32 early-fruiting nucleotide sequences from Sichuan walnut materials were performed with the maximum likelihood method. There were a total of 602 positions. All positions with gaps and missing data were eliminated, resulting in a final dataset of 562 positions. The ML tree with the highest log likelihood (−1607.82) revealed two obvious groups: one including materials of *J. regia*, which fruits 1 year after grafting, and another including materials of *J. sigillata*, which fruits >3 years after grafting. The early-fruiting gene fragment divided 22 walnut materials (10 walnut cultivars and 12 walnut accessions) into two groups, indicating that it was somewhat effective for distinguishing Sichuan walnut cultivars. Furthermore, 22 SSR loci were revealed to identify nine walnut cultivars. Eight cultivars were exclusively discerned by one SSR locus each: Chuanzao 1 [CUJRB307 (116) or CUJRA206a (182)], Chuanzao 2 [JSI-73 (154)], Shuangzao [CUJRB103a (123), CUJRB218 (144), JSI-71 (146), or CUJRA206a (176)], Shimianju [ZMZ11 (138)], Meigupao [CUJRB218 (149), CUJRB103a (151), or CUJRA206a (190)], Muzhilinhe [CUJRB220 (136), ZMZ11 (147), CUJRC310 (156), or JSI-73 (166)], Maerkang [CUJRA124 (154), CUJRB218 (159), or CUJRA123 (182)], Yanyuanzao [CUJRA124 (150) or CUJRA206a (192)]. The Shuling cultivar was identified by the combination of ZMZ11 (148) and other SSR loci, which distinguished and excluded the Chuanzao 1 and Yanyuanzao cultivars. Our results will guide the identification and breeding of Sichuan walnut cultivars.

## Introduction

**Figure 1:**
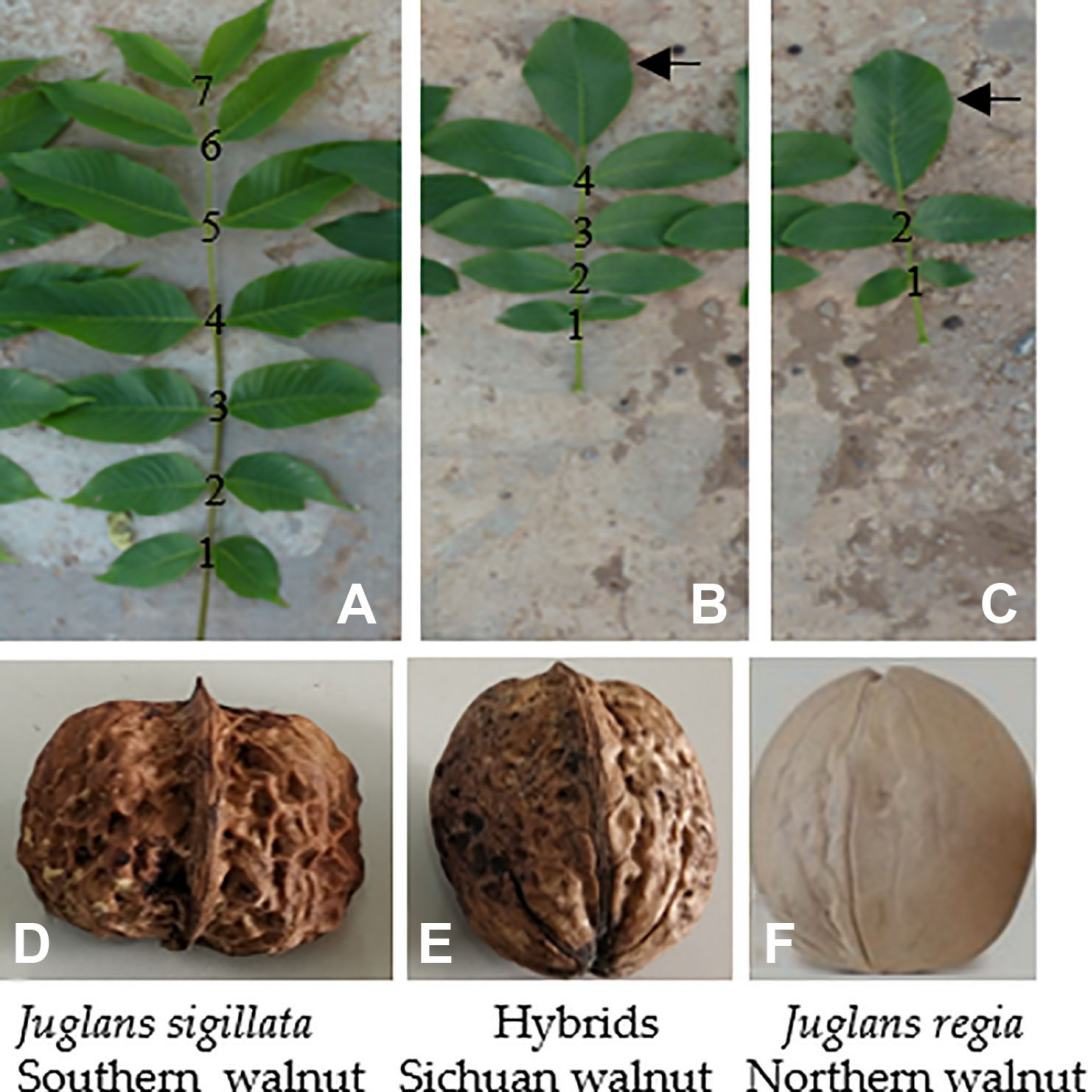
Morphological comparison of the leaves and nuts of *J. sigillata*, *J. regia* and their hybrids. *J. sigillata* has more leaflets (**A**, ~7 pairs) than *J. regia* (**C**, ~2 pairs), and their hybrids usually have ~4 pairs of leaflets **(B)**. The compound leaf of *J. sigillata* **(A)** has a small, double parietal lobe, while that of *J. regia* and the hybrids **(B, C)** commonly has a large, single parietal lobe (arrowed). The walnut shell of *J. sigillata* has a deep texture **(D)**, while that of *J. regia* has little texture or may even be flat **(F)**; the shells of the hybrids have intermediate textures **(E)**.

**Figure 2:**
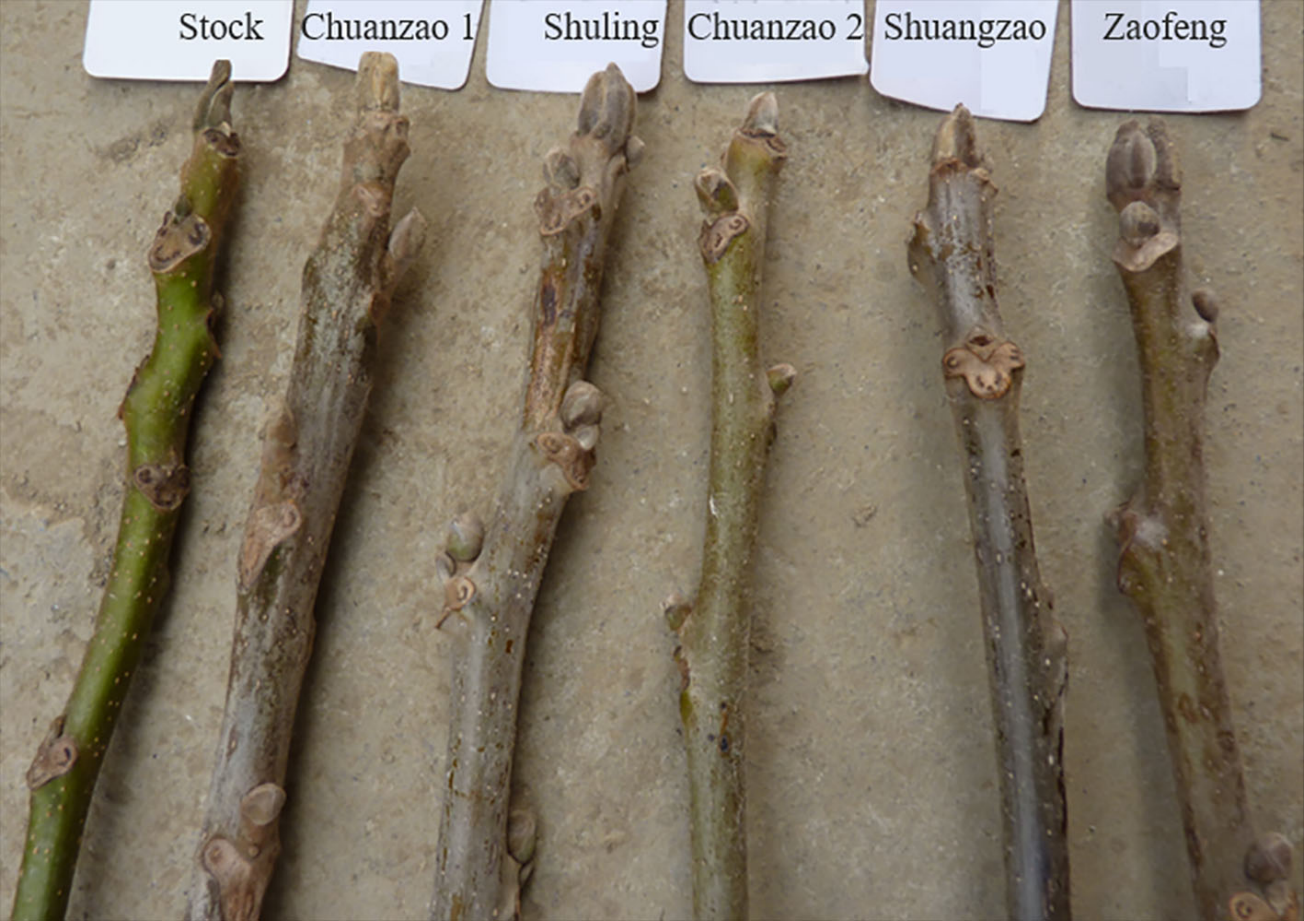
Morphological comparison of engraftment scions of Chuanzao 1, Shuling, Chuanzao 2, Shuangzao, and Zaofeng and their stocks. No distinct characters were observed for any of the engraftment scions.

Fruits are genetically very diverse groups grown in temperate, subtropical, and tropical regions and have been recognized for their human health benefits. Most of the fruits have high content of non-nutritive, nutritive, and bioactive compounds such as flavonoids, phenolics, anthocyanins, phenolic acids, and as well as nutritive compounds such as sugars, essential oils, carotenoids, vitamins, and minerals (Halasz et al., 2010; Alibabic et al., 2018; Butiuc et al., 2019). The species *Juglans regia* L. and *Juglans sigillata* Dode belong to *Juglans* in Juglandaceae, which consists of perennial woody plants that produce healthy nuts with a unique flavor and nutrient-rich composition (Kim et al., 2014). Sichuan Province is located at the boundary which is adjacent to the natural distributions of northern and southern walnuts in China. Northern walnuts (north of the Nibashan-Qinling Mountains), which grow under cold, dry, and high-sunshine conditions, and southern walnuts (south of the Erlangshan-Nibashan-Huangmaogeng-Wumengshan Mountains), which grows under warm, dry, and low-sunshine conditions (Xi and Zhang, 1996), have been introduced to Sichuan. In this area, the introduced cultivars are exposed to high-temperature, high-humidity, and low-sunshine stresses, resulting in weak trees, severe fruit drop or fruitlessness. Northern walnut is classified as *J. regia*, while southern walnut is classified as *J. sigillata*. Sichuan walnut is highly likely to be a hybrid of these two varieties. The shapes of the nuts and compound leaves of these types of walnuts are shown in **Figure 1**. *J. sigillata* has more leaflets (~7 pairs) than *J. regia* (~2 pairs), and the hybrids usually have ~4 pairs of leaflets. The compound leaf of *J. sigillata* contains a small, double parietal lobe (**Figure 1A**), while that of *J. regia* and the hybrids commonly has a large, single parietal lobe (**Figures 1B, C**). The walnut shell of *J. sigillata* has a deep texture (**Figure 1D**), while that of *J. regia* has little texture or may even be smooth (**Figure 1E**), and the shell of their hybrids has an intermediate texture (**Figure 1F**). Walnut cultivars vary greatly in quality (Pei and Lu, 2011), and tools for distinguishing among them are lacking. The engraftment scions (Chuanzao 1, Shuling, Chuanzao 2, Shuangzao, and Zaofeng) and the stock plants lack distinct characters (**Figure 2**). Therefore, a method to easily and accurately identify Sichuan walnut cultivars is greatly needed; such a method would also benefit breeders and planters. Walnut cultivars are usually distinguished based on morphology (Zenelli et al., 2005), supplemented by cytology (Tulecke et al., 1988), isozyme analysis (Fornari et al., 2001), restriction fragment-length polymorphism (RFLP) analysis (Fjellstrom and Parfitt, 1994), randomly amplified polymorphic DNA (RAPD) analysis (Ross-Davis et al., 2008), inter-simple sequence repeat (ISSR) analysis (Christopoulos et al., 2010), amplified fragment-length polymorphism (AFLP) analysis (Mozaffarian et al., 2008), simple sequence repeat (SSR) analysis (Zhou et al., 2018a; Zhou et al., 2018b; Ebrahimi et al., 2019; Wu and Gmitter, 2019; Yan et al., 2019), single-nucleotide polymorphism (SNP) analysis (You et al., 2012), and high-throughput sequencing (Wu et al., 2019; Zhang et al., 2019; Zhu et al., 2019). However, morphological studies are preliminary breeding approaches, and morphological traits are usually quantitative, meaning that they are easily controlled by the environment and thus exhibit high variability (Andersen and Lübberstedt, 2003). In addition, similar characters cannot be distinguished by morphology (Zhao, 2007), and minor differences in tree cell structure cannot be detected by cytology (Luo et al., 2018). Furthermore, cytology is dependent on the experimental conditions and procedures used as well as on the methods used to prepare slides, with large differences among individual methods (Han et al., 2018). Elucidation of effective molecular markers for the identification of walnut cultivars requires sufficient genomic data. In addition, sample collection of late-fruiting walnut (which fruits after >3 years) is time consuming. Currently available methods are insufficient for the identification of Sichuan walnut cultivars; thus, there is a great need to establish an accurate and easy way to distinguish Sichuan walnut cultivars and to determine their relationships with *J. regia* and *J. sigillata*.

Chromosome number is a basic attribute used to establish the taxonomic statuses of walnut cultivars. Species in the genus *Juglans* have 32 chromosomes (Woodworth, 1930; Hans, 1970; Mu and Xi, 1988; Tulecke et al., 1988; Mu et al., 1990). At least five molecular genetic linkage maps for walnut have been established (Fjellstrom and Parfitt, 1994; Woeste et al., 1996; Malvolti et al., 2001; Luo et al., 2015; Zhu et al., 2015). Fluorescence *in situ* hybridization (FISH) with probe-labeled chromosome ends enables accurate counting of small walnut chromosomes (Deng et al., 2019; Liu and Luo, 2019; Luo and Liu, 2019a). Karyotype analysis aids in chromosome physical map construction and chromosome assembly (Hizume et al., 2002; Luo et al., 2017; Luo et al., 2018; Pereira et al., 2018), thereby providing a guide for distinguishing Sichuan walnut cultivars to a certain degree. However, no walnut cultivars have been subjected to FISH analysis. Furthermore, DNA sequencing is much more sensitive than FISH to species differences. Early fruiting, which is a dominant character controlled by at least two alleles, is an important attribute of walnut (Xi, 1987). Early fruiting walnut cultivars are those that fruit within three years after grafting. Sequence variation in early fruiting genes occurs in response to environmental stress. The harsh northern environment favours accelerated reproduction (Charrier et al., 2011); hence, early-fruiting genes are commonly found in northern walnut (*J. regia*) but seldom found in southern walnut (*J. sigillata*). Sichuan walnuts, as hybrids, have both early-fruiting (<3 years) and late-fruiting (>3 years) characteristics. Artificial hybrids, such as Chuanzao 1 (Pu et al., 2011) and Shuangzao (Xiao et al., 2013), and natural hybrids, such as Shimianju and Yanyuanzao (Sun et al., 2011) fruit 1 year after grafting. Natural hybrids, such as Meigupao and Muzhilinhe (Wang et al., 2017) do not fruit until >3 years after grafting or planting. Early-fruiting genes have high heritability (Ye et al., 2010). The SCAR marker linked to early bearing genes in the walnut was developed by Niu et al. (2008), and its terminal sequence was cloned by Zhu et al. (2011). The early-fruiting genes *AGAMOUS*, *APETALA3*, *Ap1,* and *LFAFY* in *J. regia* were cloned and their expression analyzed by Breton et al. (2004), Ye and Niu (2011; 2012). However, no early-fruiting gene DNA sequences have been identified to distinguish Sichuan walnut cultivars. Analysis of SSRs, which are repeated units of 1 to 6 nucleotides, is a rapid, accurate, and effective technique that has been widely and successfully used to distinguish numerous walnut cultivars. Molecular studies on SSRs began to increase in the late 2000s. In 2016, the number of SSR loci identified in *Juglans* reached 15,000 (Bernard et al., 2018a), and this number has continued to increase (Shi et al., 2016; Ebrahimi et al., 2017; Bernard et al., 2018b; Chen et al., 2018; Zhou et al., 2018a; Zhou et al., 2018b; Ebrahimi et al., 2019; Chen et al., 2019; Wu et al., 2019). Although SSRs have been widely used for walnut identification, Sichuan walnut cultivars have not been well distinguished.

A total of 22 Sichuan walnut materials were used in this study. The karyotypes of four cultivars were analyzed in detail by FISH. The early-fruiting gene nucleotide sequences of 22 walnut materials (10 walnut cultivars and 12 walnut accessions) were subjected to evolutionary analysis, and nine walnut cultivars were subjected to SSR analysis. This work aimed to distinguish Sichuan walnut cultivars and examine their relationships with *J. regia* and *J. sigillata* with the goal of providing a guide for the identification and breeding of Sichuan walnut cultivars.

## Materials and Methods

In total, 10 walnut cultivars and 12 walnut accessions (belonging to *J. regia* or *J. sigillata*) were collected in Sichuan Province, China, for this study (**Table 1**). These materials included eight certified elite cultivars (Chuanzao 1, Chuanzao 2, Shuangzao, Shuling, Zaofeng, Shimianju, Muzhilinhe, and Yanyuanzao). Among these, there are five artificial hybrid cultivars, including ① *J. regia* L. ‘Chuanzao 1’ = (*J. regia* L. × *J. sigillata* Dode ‘Yunxin7926’) × (*J. regia* L. ‘Xiazao’) ‘Chuanzao 1’, ② *J. regia* L. ‘Chuanzao 2’ = (*J. regia* L. × *J. sigillata* Dode ‘Yunxin7926’) × (*J. regia* L. ‘Shahe’) ‘Chuanzao 2’, ③ *J. regia* L. ‘Shuangzao’ = (*J. regia* L. × *J. sigillata* Dode ‘Yunxin7926’) × (*J. regia* L. ‘Xiazao’) ‘Shuangzao’, ④ *J. regia* L. ‘Shuling’ = (*J. regia* L. × *J. sigillata* Dode ‘Yunxin7926’) × (*J. regia* L. ‘Xiazao’) ‘Shuling’, and ⑤ *J. regia* L. ‘Zaofeng’ = (*J. regia* L. × *J. sigillata* Dode ‘Yunxin7926’) × (*J. regia* L. ‘Xiazao’) ‘Zaofeng’. The other seventeen walnut materials were likely natural hybrids.

**Table 1:** The 22 materials used in this study, including 10 cultivars and 12 accessions.

Walnut material	License number	Species	GenBank accession number
Chuanzao 1_1	Chuan S-SV-JSJR-001-2012	*Juglans regia* L. ‘Chuanzao 1'	MN548314
Chuanzao 1_2			MN548322
Chuanzao 2	Chuan S-SC-JSJR-001-2016	*Juglans regia* L. ‘Chuanzao 2'	MN548311
Shuangzao_1	Chuan R-SC-JSJR-002-2009	*Juglans regia* L. ‘Shuangzao'	MN548308
Shuangzao_2			MN548316
Shuling	Chuan R-SV-JSJR-003-2007	*Juglans regia* L. ‘Shuling'	MN548335
Zaofeng_1	Chuan R-SC-SJR -008-2013	*Juglans regia* L. ‘Zaofeng'	MN548325
Zaofeng_2			MN548326
Shimianju	Chuan R-SC-JS-035-2011	*Juglans regia* L. ‘Shimianju'	MN548307
Muzhilinhe	Chuan R-SC-JR-002-2015	*Juglans sigillata* Dode ‘Muzhilinhe'	MN548332
Yanyuanzao_1	Chuan S-SC-JR-003-2009	*Juglans regia* L. ‘Yanyuanzao'	MN548306
Yanyuanzao_2			MN548309
Maerkang	–	*Juglans sigillata* Dode ‘Maerkang'	MN548330
Meigupao	–	*Juglans sigillata* Dode ‘Meigupao'	MN548331
4	–	*Juglans sigillata* Dode	MN548327
5	–	*Juglans sigillata* Dode	MN548317
6	–	*Juglans sigillata* Dode	MN548333
9_1	–	*Juglans sigillata* Dode	MN548318
9_2			MN548329
10	–	*Juglans sigillata* Dode	MN548337
16_1	–	*Juglans regia* L.	MN548315
16_2			MN548319
16_3			MN548320
20_1	–	*Juglans regia* L.	MN548312
20_2			MN548313
32	–	*Juglans sigillata* Dode	MN548334
42	–	*Juglans regia* L.	MN548310
45	–	*Juglans sigillata* Dode	MN548321
46_1	–	*Juglans sigillata* Dode	MN548328
46_2			MN548336
50_1	–	*Juglans regia* L.	MN548323
50_2			MN548324

### FISH Analysis

Four Sichuan walnut cultivars, namely, Chuanzao 1, Muzhilinhe, Maerkang, and Yanyuanzao, were used in this experiment. Nuts from these cultivars were germinated in wet sand at room temperature. When the lateral roots reached a length of ~2 cm, their tips were cut off, treated with nitrous oxide, and stored in 75% alcohol. Chromosome slides were prepared by digesting meristems with cellulase and pectinase (2:1), stirring the meristems, and dropping the meristems in suspension onto slides. An Olympus CX23 microscope (Olympus, Japan) was used to examine the air-dried slides. A 5S rDNA sequence (41 bp) (Luo et al., 2017) and a chromosome-end repeat oligonucleotide (AG_3_T_3_)_3_ (21 bp) (Qi et al., 2002) were used for FISH analysis. The two probes were first tested in *Juglans* species. The oligonucleotide probes were synthesized by Sangon Biotech Co., Ltd. (Shanghai, China). The synthetic oligonucleotides were 5′ end-labeled with 6-carboxyfluorescein (6-FAM) or 6-carboxytetramethylrhodamine (6-TAMRA). The synthesized probes were diluted using a 1× TE solution, maintained at a concentration of 10 μM and then stored at −20°C.

Furthermore, well-spread slides were subjected to chromosome fixation, dehydration, and denaturation before being dehydrated again and then hybridized with the denatured probe mixture at 37°C for 2 h. Subsequently, the chromosomes on the slides were washed, counterstained, and finally examined under an Olympus BX63 fluorescence microscope coupled to a Photometric SenSys Olympus DP70 CCD camera (Olympus, Japan). The raw images were examined in Photoshop version 7.1 (Adobe Systems Incorporated, San Jose, CA, USA). The chromosome nomenclature was based on length from the longest chromosome to the shortest chromosome. The chromosome ratio was calculated (over 3 metaphases) by dividing the length of the longest chromosome by the length of the shortest chromosome.

### Early-Fruiting Gene Analysis

We examined 22 Sichuan walnut materials, including 10 cultivars and 12 accessions (**Table 1**). Total genomic DNA extraction was performed as described by Doyle and Doyle (1987), with a slight change. The early-fruiting gene was amplified with the forward primer 5′-TTTGTTGTTAGACTGAATGC-3′ and the reverse primer 5′-GTGGATTTAAGGAAGGTTTG-3′. The primers were designed based on known early-fruiting sequences described by Ye et al. (2010). Each PCR (50 μL) contained 15 μL of polymerase mix, 1 mM each primer, and ~ 20 ng of template DNA complemented with ddH_2_O. The amplification protocols included 1 cycle of 5 min at 94°C; 30 cycles of 30 s at 94°C, 30 s at 62°C, and 30 s at 72°C; and 1 cycle of 10 min at 72°C. The PCR products were visualized on 0.8% agarose gels, purified with an ENZA™ gel extraction kit (Omega, Georgia, USA), and then sequenced by the Beijing Genomics Institute (Beijing, China).

The sequences outside the primers were removed from the raw sequence data. The bidirectional sequences were matched in DNAMAN 6.0.3.99 (http://www.lynnon.com/). Thirteen materials had a single sequence, eight materials had two sequences, and one material had three sequences. Therefore, the 22 materials included 32 sequences, and these clean sequences were submitted to the National Center of Biotechnology Information (NCBI) and given GenBank accession numbers ranging from MN548306-MN548337 (**Table 1**). Subsequently, evolutionary analyses were conducted in MEGA X (Kumar et al., 2018). The evolutionary history of the early-fruiting gene was inferred by using the maximum likelihood method and the Tamura-Nei model (Tamura and Nei, 1993). The initial trees used for the heuristic search were obtained automatically by applying the Neighbor-Join and BioNJ algorithms to a matrix of pairwise distances estimated using the maximum composite likelihood (MCL) approach and then selecting the topology with the superior log likelihood value. The tree was drawn to scale, with the branch lengths indicating the number of substitutions per site. The codon positions included were 1st+2nd+3rd+noncoding.

### SSR Analysis

Nine Sichuan walnut cultivars, namely, Chuanzao 1, Chuanzao 2, Shuangzao, Shuling, Shimianju, Meigupao, Muzhilinhe, Maerkang, and Yanyuanzao, were used for SSR analysis. Total genomic DNA extraction was performed as described by Doyle and Doyle (1987), with a slight change. Each PCR (15 μL) contained 5 μL of polymerase mix, 1 mM each primer, and ~20 ng of template DNA complemented with ddH_2_O. The amplification protocols included 1 cycle of 4 min at 95°C; 25 cycles (or 30 cycles depending on the primers) of 1 min at 94°C, 1 min at 55°C, and 1 min at 72°C; and 1 cycle of 7 min at 72°C. The forward primers were labeled with 6-FAM, HEX, and ROX.

Based on the genomic data for walnut (*J. regia* and *J. sigillata*) available from the NCBI (Martinez-Garcia et al., 2016; Luo and Liu, 2019b), 21 pairs of primers (**Table 2**) were obtained after two rounds of screening. Then, the fluorescent amplification products were sequenced on an ABI 3730 DNA analyser (Applied Biosystems), and allele sizing was performed using Gene Mapper™ 4.1 software (Applied Biosystems). The obtained allele sizes and relative peak heights were analyzed in Excel 2019.

**Table 2:** The 21 SSR loci distinguish Sichuan walnut materials used in this study.

SSR loci	Repeat motif	Product size (bp)	Forward (F) and reverse (R) primers (5’-3’)
CUJRA123	(AC)_12_	193	F-TTGGTCTCTTCTTTCCTCTATG R-TCGAACGTACAATAACGTACAG
CUJRA124	(GT)_13_	151	F-CGTTGCCTGAACAAGTAAGAT R-GAAGGAGGCTAACTCCCTATG
CUJRA206a	(AC)_16_	197	F-GCCGAGAGAGGAAGAGAGACT R-CGACTACAGGGACCAATCAAC
CUJRB012	(AG)_15_	102	F-ACTCATCAAGATCCCCGACTAC R-CCACATCGTCTGGGTTCAT
CUJRB103a	(TC)_17_	148	F-CATGCTATGGACTACCTCCTCR-AAGAGAGACGAACGAAGAGTG
CUJRB218	(GA)_13_	159	F-CTAGCGTCGAAGAAGAAGATG R-TTGTTTCTCCTCTGTCATGTTT
CUJRB220	(TC)_25_	163	F-AGCATGTATAGGCCAATGATG R-TCGTTCTATCTACAAGCACTCG
CUJRB305	(GA)_19_	132	F-GCTGCTTTATTAGCCATGATC R-GGTTCAATGTGCAACAAGAG
CUJRB307	(GA)_23_	146	F-CTGGGCTGAAGGAGAATC R-TTGGATGTCTGCTTTTTTAGAG
CUJRB317	(TC)_17_	125	F-TGCCCACTAACCCTAACC R-GAGAAAAAGAATGGCTGTATTG
CUJRC310	(TTG)_7_	142	F-GCTGTTAGTGGAATCCCAACT R-TAAACGTGATCGAAGTGAAATG
CUJRD204a	(CTC)_4_	154	F-CAGCCAATCTTCTTCTGCTTC R-GAGACCTACGACCACGATCAC
JH2753	(GCT)_6_	188–212	F-CAGTTTTGGCCAGCTGCAAT R-TGTGCCCATGCTAAGACTGG
JUG-13	(GGA)_5_	240–250	F-GAAGAGACTCCGTTGCCACA R-ACTCCGTCGTTTCCCTGAAC
JSI-15	(TC)_6_	180–200	F-ATGAGAGCCAGCCAACAGAC R-CGAGCGAGCAAGAGAGAGAG
JSI-63	(GATCG)_5_	180–200	F-TCCGGACAACTCCTCATCCT R-CTCTCCGCCGAGTCATGTAC
JSI-71	(GCAGTA)_8_	135–155	F-AGCTAGCTCTCAAACAACAAGC R-ACAAACATGGCAACCTTCGTG
JSI-73	(TGCTCG)_5_	160–175	F-AGCTCAACGGTCAAGGAAGG R-GGAGAGAGAGAGCTCGGCTA
JRE-28	(AAG)_5_	150–170	F-CCGGGAAGCTCAGTTCAAGA R-GGTTCTTCCGCAGTTGGTCT
JRE-46	(GAA)_18_	190–220	F-GCCTCTCCTCGTGCTCATTT R-ACTCGCTACTTTTCAGGCCC
ZMZ11	(CTG)_5_	160	F-CCAGAACCAGGAGCCAGCAA R-GACCATCGGCCCGAAAGTAA

## Results

### FISH Analysis

**Figure 3:**
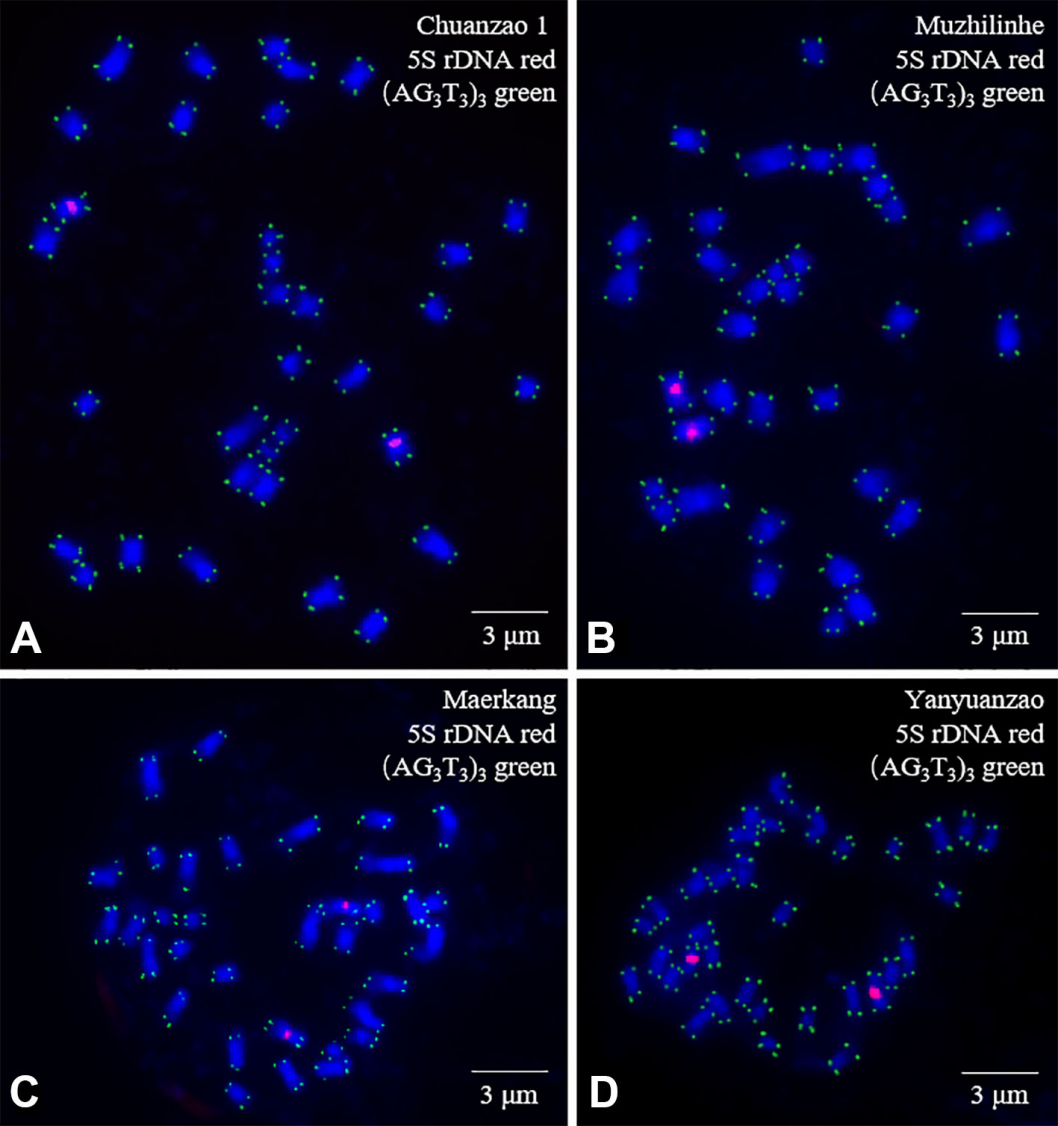
Mitotic metaphase chromosomes of four walnut cultivars: **(A)** Chuanzao 1, **(B)** Muzhilinhe, **(C)** Maerkang, and **(D)** Yanyuanzao. Red represents 5S rDNA, while green represents (AG_3_T_3_)_3_. The blue chromosomes were counterstained by DAPI. Scale bar = 3 μm.

**Figure 4:**
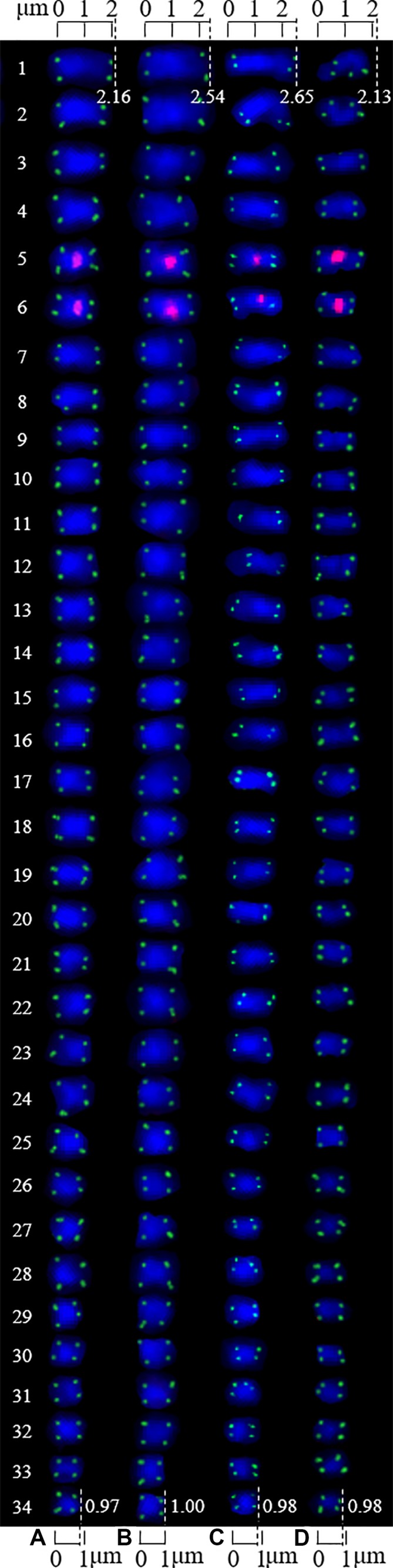
Chromosomes from **Figure 3** presented individually. The chromosomes were aligned by length from longest to shortest. The left number represents the chromosome number, while the top/bottom number represents the chromosome length. **(A)** Chuanzao 1, 2.16–0.97 μm; **(B)** Muzhilinhe, 2.54–1.00 μm; **(C)** Maerkang, 2.65–0.98 μm; **(D)** Yanyuanzao, 2.13–0.98 μm.

The mitotic metaphase chromosomes of four walnut cultivars (Chuanzao 1, Muzhilinhe, Maerkang, and Yanyuanzao) after FISH are shown in **Figure 3**. Furthermore, the chromosomes shown in **Figure 3** are presented individually in **Figure 4**. A total of 34 chromosomes were counted in all four walnut cultivars. 5S rDNA oligonucleotides were located in the proximal regions of two chromosomes (5 and 6) in each cultivar (red), while (AG_3_T_3_)_3_ was located at both ends of each chromosome in each cultivar (green). The existence of a signal at both chromosome ends ensured accurate chromosome counts. The fluorescence intensity of 5S rDNA was stronger than that of (AG_3_T_3_)_3_. The fluorescence intensity of 5S rDNA varied, but that of (AG_3_T_3_)_3_ was consistent among the four cultivars. Among the cultivars, the Yanyuanzao cultivar exhibited the strongest fluorescence intensity of 5S rDNA, the Maerkang cultivar exhibited the weakest fluorescence intensity, and the other two cultivars (Chuanzao 1 and Muzhilinhe) exhibited intermediate intensity. There were no number differences or obvious intensity differences in the fluorescence signals among the four cultivars. Since 5S rDNA and (AG_3_T_3_)_3_ were insufficient for identifying Sichuan walnut cultivars, further methods for detection were necessary.

The chromosomes of each cultivar were aligned by length in one column from longest (#1) to shortest (#34). The chromosome lengths of the Chuanzao 1, Muzhilinhe, Maerkang, and Yanyuanzao cultivars were 2.16 to 0.97 μm, 2.54 to 1.00 μm, 2.65 to 0.98 μm, and 2.13 to 0.98 μm, respectively. Since these lengths were less than 3 μm, the chromosomes of these four walnut cultivars are small chromosomes. The chromosomes of the Muzhilinhe and Maerkang cultivars were slightly larger than those of the other cultivars (Chuanzao 1 and Yanyuanzao). The chromosome ratios of the Chuanzao 1, Muzhilinhe, Maerkang, and Yanyuanzao cultivars were 2.23, 2.54, 2.70, and 2.17, respectively, indicating the following order in terms of chromosome asymmetry: Maerkang> Muzhilinhe> Chuanzao 1> Yanyuanzao. Due to the hidden centromere in a large proportion of the chromosomes, the long arm and short arm were not determined, and further karyotype analysis was not carried out. These limited karyotype data contributed little to the identification of the walnut cultivars.

### Early-Fruiting Gene Analysis

**Figure 5:**
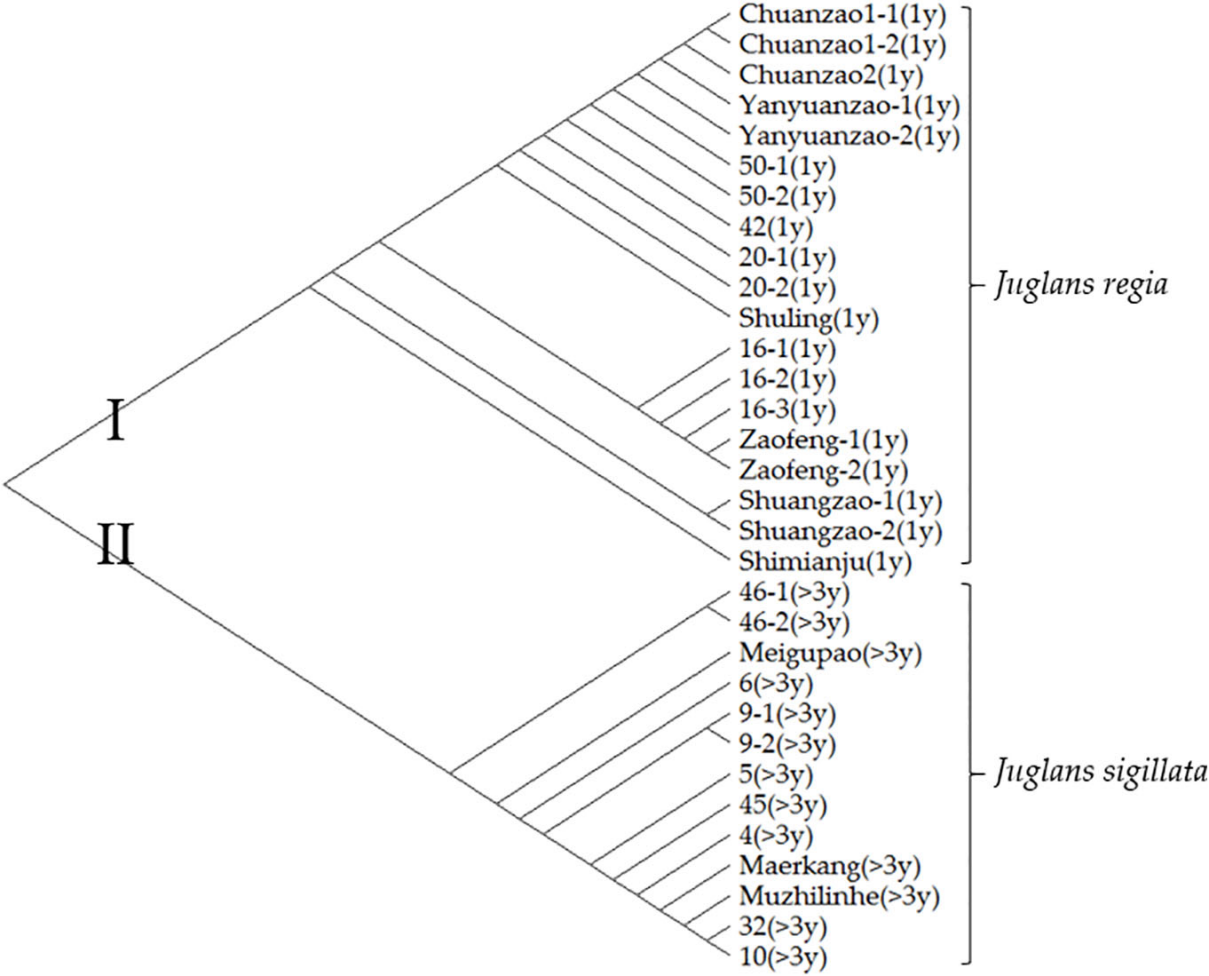
Evolutionary analysis involving thirty-two early-fruiting nucleotide sequences from 22 walnut materials was performed with the maximum likelihood method. Evolutionary history was inferred by using the maximum likelihood method and the Tamura-Nei model. Evolutionary analyses were conducted in MEGA X. The information in parentheses represents the number of years after planting before fruiting occurs.

To further distinguish Sichuan walnut cultivars, evolutionary analysis involving thirty-two early-fruiting nucleotide sequences from 22 walnut materials was performed with the maximum likelihood method; the results are shown in **Figure 5**. The tree with the highest log likelihood (−1607.82) is shown. There were a total of 602 positions. All positions with gaps and missing data were eliminated, resulting in a final dataset of 562 positions. There were two obvious groups in the tree. The first group included nineteen early-fruiting nucleotide sequences covering eleven *J. regia* materials with fruiting one year after grafting. The second group included thirty early-fruiting nucleotide sequences covering the other eleven *J. sigillata* materials with fruiting >3 years after grafting or planting. The seven cultivars Chuanzao 1, Chuanzao 2, Yanyuanzao, Shuling, Zaofeng, Shuangzao, and Shimianju belonged to the first group, while the other three cultivars (Meigupao, Maerkang, and Muzhilinhe) belonged to the second group. Since the early-fruiting gene fragment divided the ten walnut cultivars into two groups, it was able to distinguish the cultivars to some degree, but further methods of detection were needed.

### SSR Analysis

**Figure 6:**
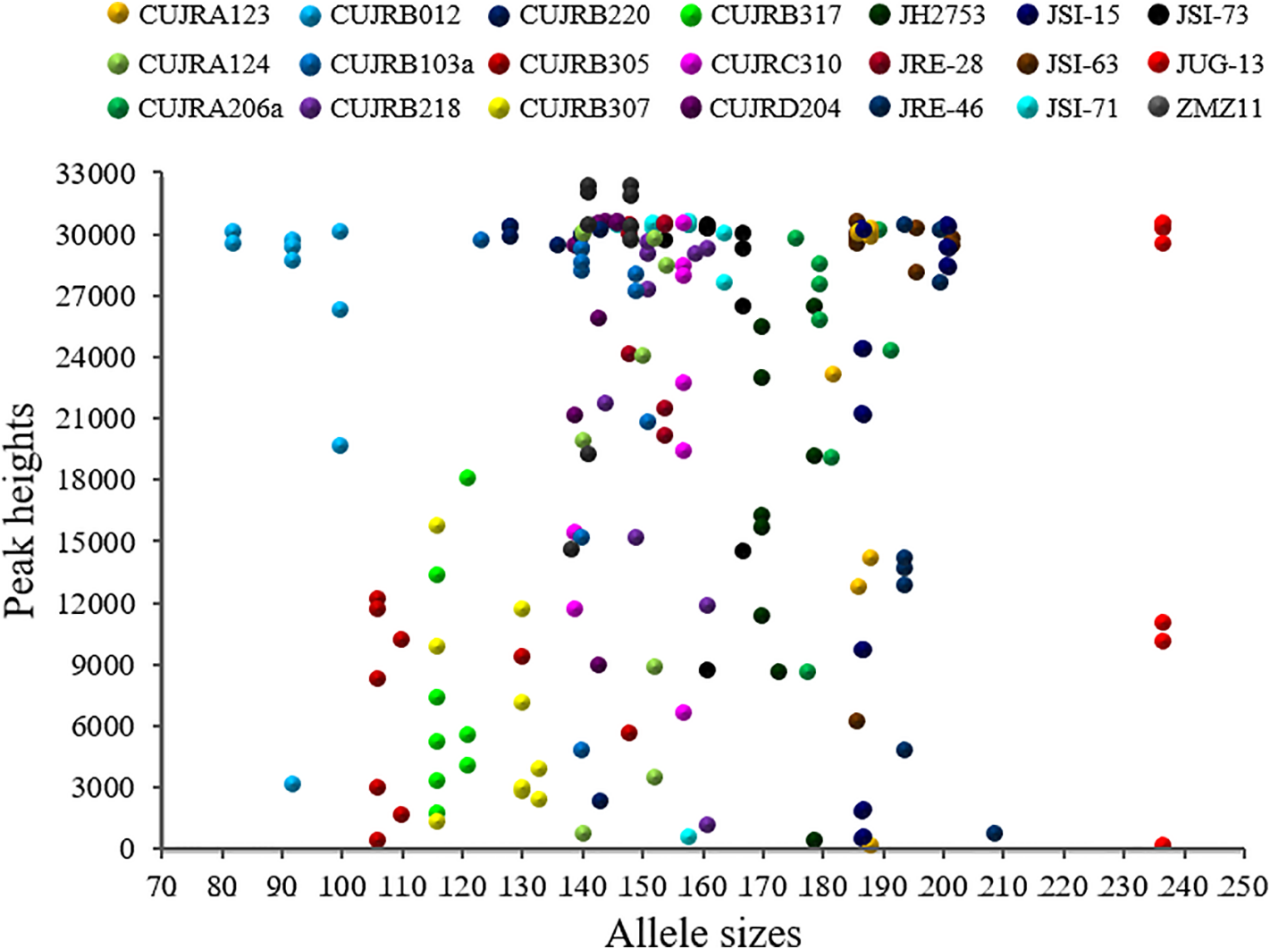
Scatter diagram of 21 SSR loci detected in the nine walnut cultivars. The SSR loci are represented by different colors and symbols. The x-axis indicates allele size, while the y-axis indicates relative peak height. The allele sizes ranged from 82 to 237 nucleotides, while the relative peak heights ranged from 201 to 32,722.

To effectively distinguish the walnut cultivars, we further developed 21 SSR loci to detect nine walnut cultivars. A scatter diagram of these 21 SSR loci is provided in **Figure 6**. The allele sizes ranged from 82 to 237 nucleotides, while the relative peak heights ranged from 201 to 32,722. Most alleles were 135 to 200 nucleotides in length, while no alleles were 210 to 235 in size. Among the alleles, 3% were less than 100 nucleotides, 9% were greater than 200 nucleotides, and 87% were between 100 and 200 nucleotides in length. Half of the relative peak heights ranged from 25,000 to 32,722, while the others ranged from 200 to 25,000. Among these relative peak heights, 21% were less than 10,000, 15% were from 10,000 to 20,000, 17% were from 20,000 to 30,000, and 47% were greater than 30,000.

**Figure 7:**
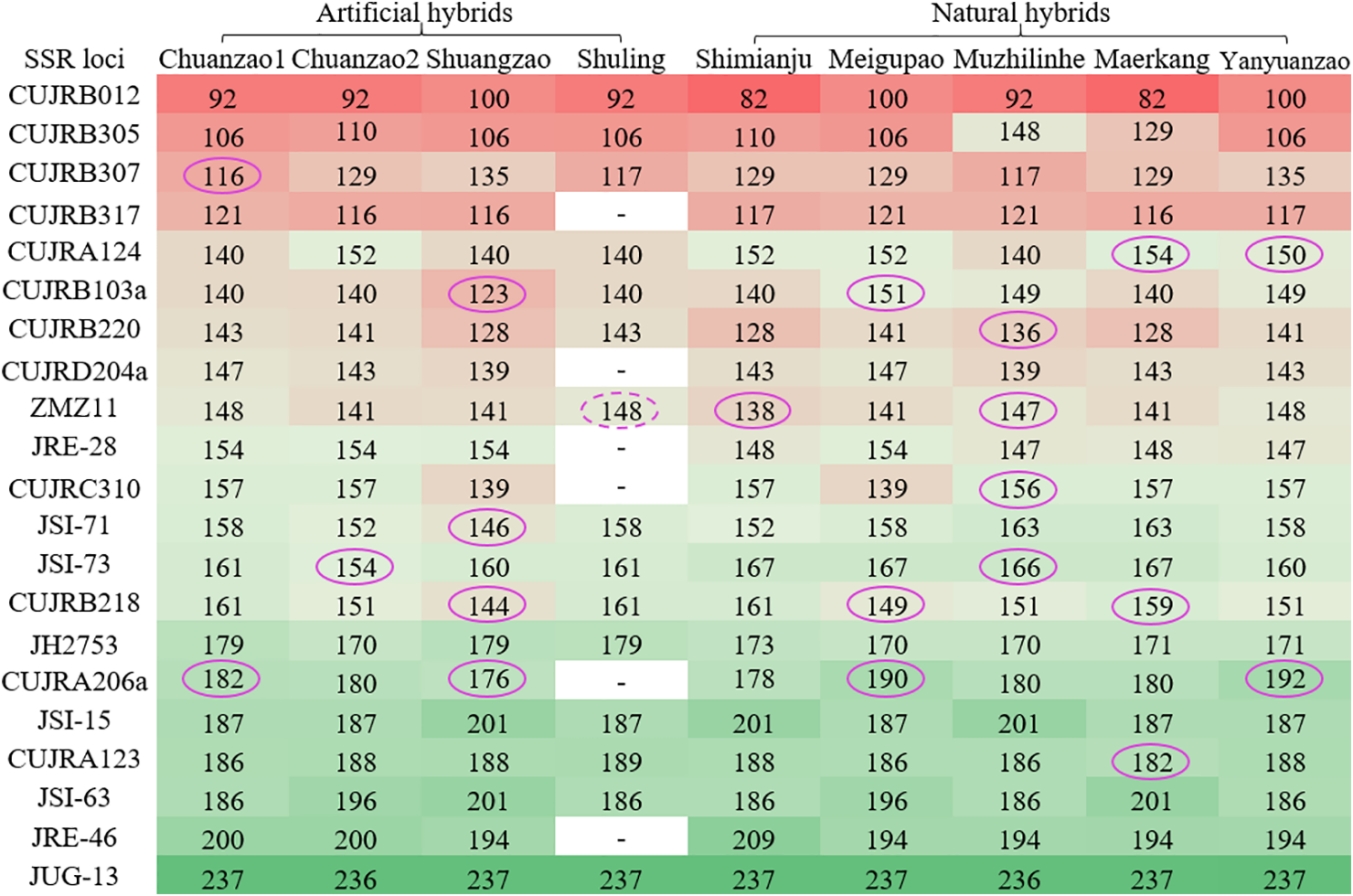
Allele sizes of 21 SSR loci of the four artificial hybrid cultivars and five natural hybrid cultivars. The SSR loci were aligned based on the allele size of the Chuanzao 1 cultivar from smallest to largest. Color gradients of red, blue, and green were used to mark all the allele sizes in Excel 2019. Red represents small allele sizes, green represents large allele sizes, and light blue represents intermediate allele sizes. Six SSR loci of the Shuling cultivar were not obtained, as shown by “-” in the column for this cultivar. The allele sizes in purple ovals are those that can be used to discern the cultivars (except for allele size 148 in the dashed oval, because Shuling can be discerned by the combination of ZMZ11 (148) and other SSR loci, which distinguish and exclude Chuanzao 1 and Yanyuanzao.

Furthermore, the allele size of each of the 21 SSR loci is shown in **Figure 7**. In total, 183 allele sizes were obtained, excluding six missing allele sizes (white) for the Shuling cultivar. The SSR loci were aligned based on the allele size of the Chuanzao 1 cultivar from smallest (red) to largest (blue). To precisely and easily discern the four artificial hybrid cultivars and five natural hybrid cultivars, repeated allele sizes and relative peak heights less than 14,000 were filtered out. Subsequently, several effective allele sizes were maintained, as indicated by the purple ovals. Eleven SSR loci helped discern eight of the cultivars (excluding the Shuling cultivar); these SSR loci were CUJRB307, CUJRA124, CUJRA123a, CUJRB220, ZMZ11, CUJRC310, JSI-71, JSI-73, CUJRB218, CUJRA206a, and CUJRA213. Each of the eight cultivars (Chuanzao 1, Chuanzao 2, Shuangzao, Shimianju, Meigupao, Muzhilinhe, Maerkang, and Yanyuanzao) was exclusively discerned by one SSR locus. Due to the lack of allele sizes, the Shuling cultivar was discerned by a repeat allele size of 148 nucleotides, as shown by the dashed oval. Hence, it was possible to discern the Shuling cultivar with a combination of ZMZ11 and other SSR loci, which distinguished and excluded the Chuanzao 1 and Yanyuanzao cultivars.

**Figure 8:**
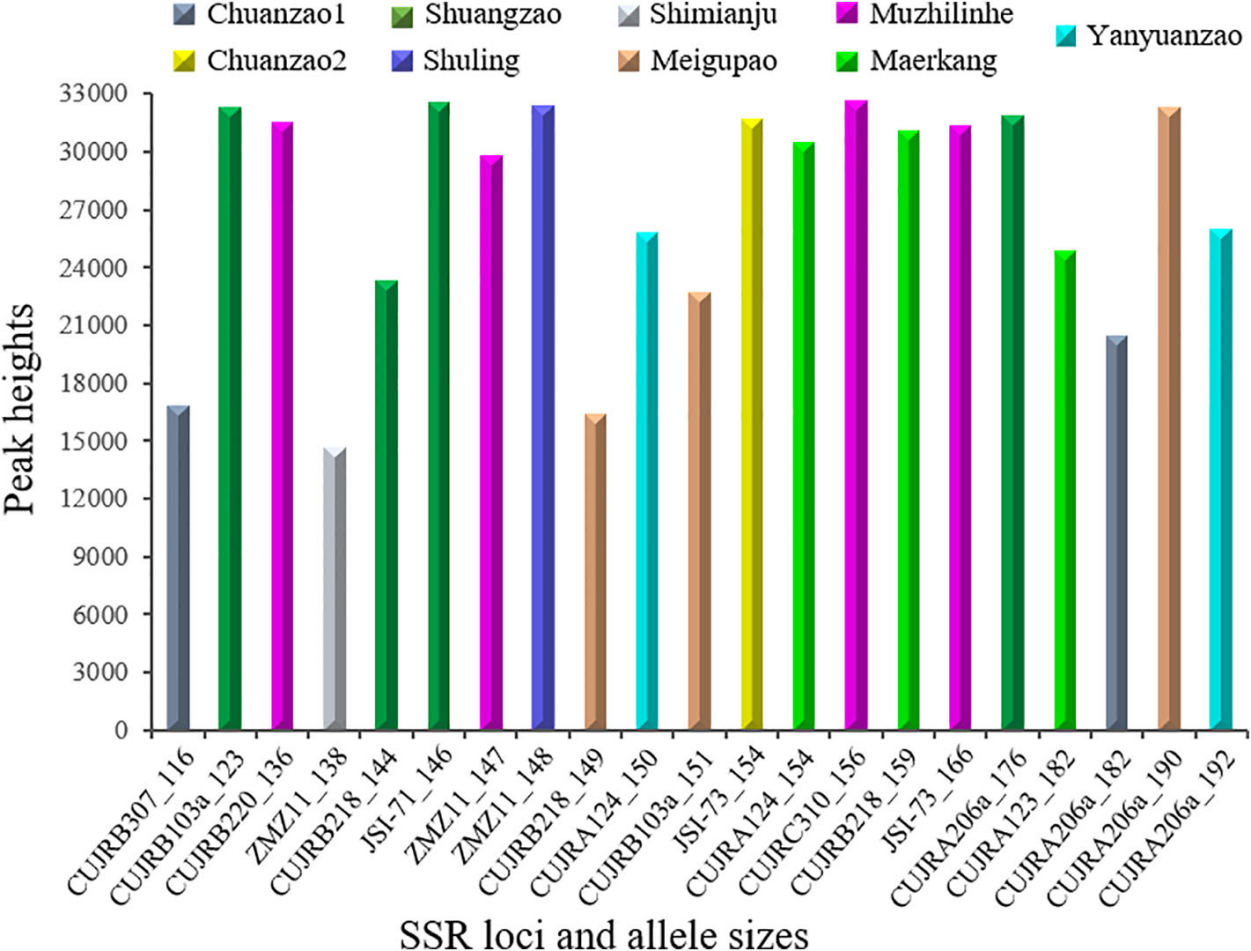
Nine cultivars were discerned by 21 selected SSR allele sizes. The x-axis indicates SSR locus and allele size, while the y-axis indicates relative peak height. The allele size ranged from 116 to 192 nucleotides, while the relative peak height ranged from 14,643 to 32,607. The Chuanzao 1 cultivar was discerned by CUJRB307 (116) or CUJRA206a (182); Chuanzao 2 was discerned by JSI-73 (154); Shuangzao was discerned by CUJRB103a (123), CUJRB218 (144), JSI-71 (146), or CUJRA206a (176); Shimianju was discerned by ZMZ11 (138); Meigupao was discerned by CUJRB218 (149), CUJRB103a (151), or CUJRA206a (190); Muzhilinhe was discerned by CUJRB220 (136), ZMZ11 (147), CUJRC310 (156), or JSI-73 (166); Maerkang was discerned by CUJRA124 (154), CUJRB218 (159), or CUJRA123 (182); Yanyuanzao was discerned by CUJRA124 (150) or CUJRA206a (192); and Shuling was discerned by the combination of ZMZ11 (148) and other SSR loci, which distinguished and excluded Chuanzao 1 [CUJRB307 (116) or CUJRA206a (182)] and Yanyuanzao [CUJRA124 (150) or CUJRA206a (192)].

**Figure 8** further shows the nine cultivars discerned by the 21 selected SSR allele sizes from eleven SSR loci summarized in **Figure 7**. The allele sizes ranged from 116 to 192 nucleotides, while the relative peak heights ranged from 14,643 to 32,607. The Chuanzao 1 cultivar was exclusively discerned by CUJRB307 (116) or CUJRA206a (182); the Chuanzao 2 cultivar was exclusively discerned by JSI-73 (154); the Shuangzao cultivar was exclusively discerned by CUJRB103a (123), CUJRB218 (144), JSI-71 (146), or CUJRA206a (176); the Shimianju cultivar was exclusively discerned by ZMZ11 (138); the Meigupao cultivar was exclusively discerned by CUJRB218 (149), CUJRB103a (151), or CUJRA206a (190); the Muzhilinhe cultivar was exclusively discerned by CUJRB220 (136), ZMZ11 (147), CUJRC310 (156), or JSI-73 (166); the Maerkang cultivar was exclusively discerned by CUJRA124 (154), CUJRB218 (159), or CUJRA123 (182); the Yanyuanzao cultivar was exclusively discerned by CUJRA124 (150) or CUJRA206a (192); and the Shuling cultivar was discerned by the combination of ZMZ11 (148) and other SSR loci, which distinguished and excluded the Chuanzao 1 [CUJRB307 (116) or CUJRA206a (182)] and Yanyuanzao [CUJRA124 (150) or CUJRA206a (192)] cultivars.

## Discussion

Rapid and accurate identification of Sichuan walnut cultivars is particularly important in grafting propagated nut tree species both for practical breeding purposes and for cultivar proprietary rights protection. In this study, we used FISH, early-fruiting gene analysis and SSR analysis to identify Sichuan walnut cultivars. We will discuss the results with respect to the following two aspects: *i*) distinguishing Sichuan walnut cultivars by molecular cellular inheritance and *ii*) determining the relationships of Sichuan walnuts with *J. regia* and *J. sigillata*.

### Distinguishing Sichuan Walnut Cultivars by Molecular Cellular Inheritance

**Figure 9:**
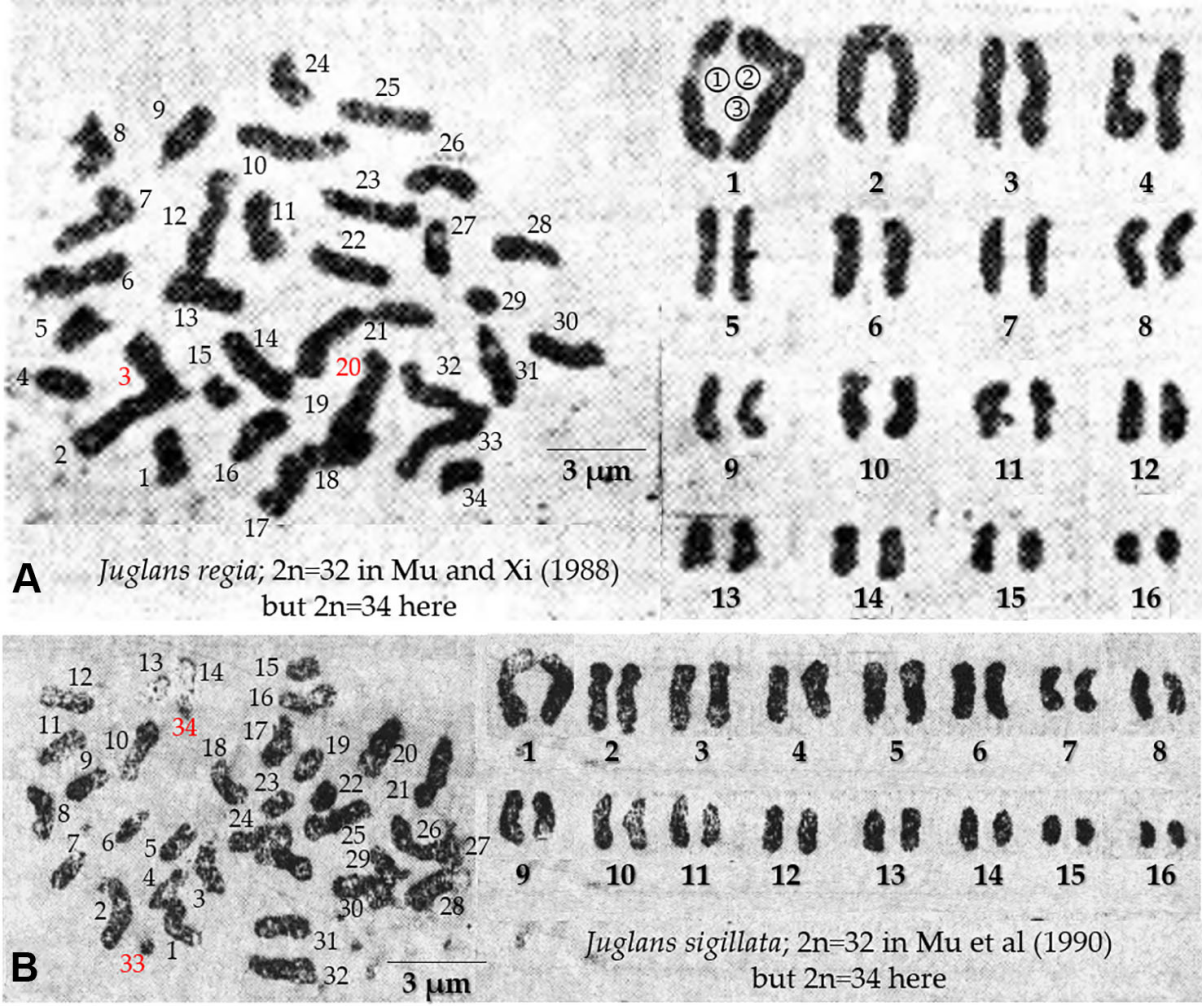
Metaphase chromosomes of *J. regia* **(A)** and *J. sigillata* **(B)**. Mu and Xi (1988) reported 2n = 32 *J. regia* chromosomes, but 2n = 34 were counted here. The first pair of chromosomes in **(A)** probably consists of three chromosomes, and the chromosomes with red numbers are likely disputed extra chromosomes. Mu et al. (1990) reported 2n = 32 *J. sigillata* chromosomes, but 2n = 34 were counted here. The chromosomes with red numbers in **(B)** are likely disputed extra chromosomes.

In contrast to morphological analysis, FISH, early-fruiting gene analysis and SSR analysis are three methods of molecular cellular inheritance analysis. The Sichuan walnut cultivars showed strong differences in morphological characters, such as leaf and nut shell characters. However, these characters are easily controlled by the environment. Therefore, we first performed FISH to distinguish Sichuan walnut cultivars. In contrast to the findings of previous studies, thirty-four chromosomes were detected by chromosome-end fluorescence signals in all four cultivars in this study. Several previous works have reported that *Juglans* has thirty-two chromosomes (Woodworth, 1930; Hans, 1970; Tulecke et al., 1988; Mu and Xi, 1988; Mu et al., 1990). The original chromosome images obtained by Hans (1970); Tulecke et al. (1988), and Woodworth (1930) have not been checked. However, thirty-four chromosomes were counted in the original image of *J. regia* (**Figure 9A**), but Mu and Xi (1988) reported thirty-two chromosomes. Similarly, thirty-four chromosomes were counted in the original image of *J. sigillata* (**Figure 9B**), whereas Mu et al. (1990) reported thirty-two chromosomes. A similar phenomenon has been observed in the *Carya* species *C. aquatica* (2n = 32), *C. dabieshanensis* (2n = 32), *C. hunanensis* (2n = 32), *C. myristiciformis* (2n = 32), *C. tonkinensis* (2n = 32), *C. illinoinensis* (2n = 34), *C. ovata* (2n = 34), *C. floridana* (2n = 64), and *C. texana* (2n = 64) (Xu, 2017). During adaptive evolution, the chromosome numbers and forms of several plants change *via* polyploidization, aneuploidization (caused by deletion, duplication, inversion, or translocation), and B chromosome generation (Schubert, 2007; Guerra, 2008; Raskina et al., 2008; Weiss-Schneeweiss et al., 2008). For example, in plants in the genera *Carex* and *Prospero*, chromosomes commonly undergo breakage and fusion (Hipp and Roalson, 2009; Chung et al., 2011; Jang et al., 2013), especially *Carex* species, in which chromosome numbers range from 6 to 46 (Roalson, 2008). Both *Trifolium subterraneum* (2n = 16) and *Trifolium israeliticum* (2n = 12) are produced by aneuploidization of *T. israeliticum* (2n = 14) (Falistocco et al., 2013). In Brassicaceae plants, a chromosome count of n = 8 can be reduced to a count of n = 7 (Mandakova and Lysak, 2008). The numbers of chromosomes vary among individuals within populations of *Phaseolus vulgaris* (Pedrosa-Harand et al., 2006). Hence, the possible reasons for the differences in walnut chromosome numbers between previous works and this study are as follows: *i*) Adaptive evolution. Aneuploidization variation in the four Sichuan walnut cultivars is likely because the chromosome numbers varied from 32 to 34. *ii*) Among-population variation. Similar to those of *P. vulgaris*, *C. illinoinensis,* and *C. ovata*, the chromosome numbers of the four Sichuan walnut cultivars probably vary among populations of individuals, and we determined the walnut cultivars to have 2n = 34. *iii*) Hybridization. Continuous hybridization has probably caused the chromosomes of the four Sichuan walnut cultivars to become mismatched and to misdivide during meiosis and mitosis because the parents have different genetic backgrounds. *iv*) Inaccurate chromosome number counts. Due to a lack of available technology for effectively discerning intact chromosomes, the chromosomes did not spread very well in previous study.

Although walnut chromosome numbers have been reported previously, the FISH technique has not yet been used to analyse walnut chromosomes. The FISH 5S rDNA oligonucleotide with two signals and (AG_3_T_3_)_3_ with an end signal on every chromosome showed almost no differences among the four walnut cultivars in this study. Similar distribution patterns have also been observed in *Citrus* species (Deng et al., 2019); *Berberis* species (Liu and Luo, 2019); and *Fraxinus*, *Syringa*, and *Ligustrum* species (Luo and Liu, 2019a). FISH probes (AG_3_T_3_)_3_ located at chromosome ends ensure accurate chromosome counts (Pereira et al., 2018), although (AG_3_T_3_)_3_ is also occasionally observed in the internal positions of chromosomes (Murray et al., 2002; Majerová et al., 2014; Vasconcelos et al., 2018). Because 5S rDNA and (AG_3_T_3_)_3_ were inadequate for identifying the Sichuan walnut cultivars, it was necessary to perform further detection. The hidden centromeres of the small chromosomes hampered further karyotype analysis. A similar phenomenon has also been observed in *Fraxinus pennsylvanica*, *Syringa oblata*, *Ligustrum lucidum*, and *Ligustrum* × *vicaryi* (Luo and Liu, 2019a) as well as in *Zanthoxylum armatum* (Luo et al., 2018). The limited karyotype data contributed little to the identification of the walnut cultivars.

It is difficult for FISH to discern interspecific and intraspecific differences (Liu and Luo, 2019; Luo and Liu, 2019a). In addition, it is rather hard to prepare well-distributed chromosomes and explore the ability of probes to identify walnut varieties. Root samples for chromosome counts should be collected from cuttings of sample trees. However, walnut plants rarely produce fat young roots; even seedlings usually grow all thin old roots. Hence, the root materials of the four walnut cultivars used in this study originated from germinated seeds. It takes approximately at least two months for walnuts to germinate, and then the lateral roots must branch from the main root. Root tips from lateral roots were used in this study. It was quite difficult to obtain even one well-spread slide. Furthermore, when potential sequences with differences between cultivars are obtained, these sequences must be converted into FISH probes. If these sequences are oligonucleotides (< 60 bp), the FISH probes are inexpensive, easy to prepare, and effective. However, if these sequences are longer, preparation of FISH probes is expensive and more time consuming. When the probes are ready, additional slides are needed to test if the probes produce signals. In addition, for visualization of the fluorescence signal, the probes must be repeatedly used in adjacent chromosomes up to 10 kbp long in metaphase (Pedersen and Linde-Laursen, 1995). Therefore, it is not easy to explore the identification ability of probes. In this study, we prepared well-spread chromosomes and developed probes located at chromosome ends and proximal regions, but the probes were not able to distinguish the walnut cultivars. Hence, it is necessary to explore more probes to identify walnut cultivars.

Furthermore, an evolutionary analysis of early-fruiting nucleotide sequences clearly revealed two groups of *J. regia* and *J. sigillata* in this study. DNA sequences of gene fragments can ordinarily be used to distinguish species in different genera; however, they are less well suited to reveal interspecies differences within the same genus and can rarely be used to discern cultivars in the same species. Previous works on early-fruiting genes have mainly concentrated on *J. regia* and have included *in vitro* cultivation (Breton et al., 2004), molecular marker analysis (Niu et al., 2008; Ye et al., 2010), and cloning and expression analysis (Niu et al., 2008; Zhu et al., 2011). Few studies on early-fruiting genes in *J. sigillata* are available. The two species, *J. regia* and *J. sigillata,* have also been identified by restriction site-associated DNA SNPs (RAD-SNPs) (Yan et al., 2019) and RAPD (Chen et al., 2007). However, the chloroplast genomes of these two species differ little (Hu et al., 2017). In addition, early-maturing walnuts are more diverse than and are distinct from normally maturing walnuts (Ebrahimi et al., 2017). Walnuts feature precocious genotypes and exhibit a tendency towards homozygosity due to self-pollination (Chen et al., 2018). Since the early-fruiting gene fragment divided the ten walnut cultivars into two groups, it was somewhat useful for recognition; however, additional methods of detection are still needed.

We further developed 21 SSR loci to detect nine Sichuan walnut cultivars in this study. Each of eight cultivars (Chuanzao 1, Chuanzao 2, Shuangzao, Shimianju, Meigupao, Muzhilinhe, Maerkang, and Yanyuanzao) was exclusively discerned by one SSR locus, and the Shuling cultivar was discerned by a combination of SSR loci. Shi et al. (2016) identified seven Sichuan walnut cultivars by selecting combinations of SSR loci from previous works (Woeste et al., 2002; Dangl et al., 2005; Ross and Woeste, 2008; Wang et al., 2011). Zhou et al. (2018a; 2018b) constructed SSR fingerprints of elite Sichuan cultivars of *J. regia* and *J. sigillata*. Although several walnut cultivars were examined by Shi et al. (2016) and Zhou et al. (2018a; 2018b), this study provided the following novel contributions: *i*) new SSR loci were developed, *ii*) each of eight cultivars was exclusively discerned by one SSR locus, and *iii*) a few cultivars were tested for the first time. In total, approximately fifty elite walnut cultivars are licensed in Sichuan Province (http://www.sclmzm.com:88/sczmz/linmuliangzhong/index.jhtml). To date, no study has distinguished all of these cultivars. The main barriers to doing so are difficulties in sample collection from all the cultivars and inadequate identification techniques. Hence, more work is needed to explore professional identification techniques for all the walnut cultivars in Sichuan. We are devoting to testing oligonucleotides probes from our SSR repeat motifs, developing more SSR loci that exclusively distinguish all the walnut cultivars in Sichuan, using additional methods such as Hi-C (High-throughput chromosome conformation capture) to construct chromosome conformation of Sichuan walnuts.

### Relationships of Sichuan Walnuts with *J. regia* and *J. sigillata*

The geographical location of Sichuan has led to the development of natural hybrids of Sichuan walnuts, while social development and human demands have led to the production of artificial hybrids of Sichuan walnuts (Xiao and Pu, 2015). It is unclear whether each hybrid Sichuan walnut belongs to one of its parent species (*J. regia* or *J. sigillata*) or represents a new taxon of *Juglans*. For example, the Shimianju cultivar was treated as *J. regia* by Sun et al. (2011); Shi et al. (2016), and Wu (2009) but as *J. sigillata* by Yan et al. (2019) and Zhou et al. (2018a; 2018b). Sichuan walnuts are intermediate between *J. regia* and *J. sigillata* in terms of their morphological characters (Xiao and Pu, 2015; Zhou et al., 2018a; 2018b). Sichuan walnuts (2n = 34) have been reported to possess two more chromosomes than *J. regia* and *J. sigillata* in previous works (Woodworth, 1930; Hans, 1970; Tulecke et al., 1988; Mu and Xi, 1988; Mu and Xi, 1990). A probable reason for this increase in chromosome number is chromosome mismatching or misdivision during meiosis and mitosis caused by multiple hybridizations between *J. regia* and *J. sigillata*, which have genetic differences. Sichuan walnuts are different from *J. regia* and *J. sigillata* in terms of their morphological characters, and their chromosome numbers seem to provide some support for their classification in a new taxon of *Juglans*. However, the evolutionary analysis of the early-fruiting gene of Sichuan walnut cultivars placed some of them within *J. regia* and some of them within *J. sigillata*, which does not support the grouping of Sichuan walnut cultivars in a new *Juglans* taxon. The SSR allele sizes of the Sichuan walnut cultivars were similar and did not support the existence of a new taxon of *Juglans*. In summary, Sichuan walnut cultivars vary greatly: some are similar to *J. regia,* some are similar to *J. sigillata*, and some are intermediate. However, these differences are not sufficient to treat these cultivars as a new *Juglans* taxon.

## Data Availability Statement

The DNA sequencing generated in this study was submitted to the National Center of Biotechnology Information (NCBI) and given GenBank accession numbers ranging from MN548306-MN548337.

## Author Contributions

Conceptualization: XL. Methodology: JC. Software: JC. Validation: XL. Formal analysis: XL. Investigation: JC. Resources: XL. Data curation: XL. Writing—original draft preparation: XL. Writing—review and editing: XL. Visualization: JC. Supervision: XL. Project administration: XL. Funding acquisition: XL. All authors consented to this submission.

## Funding

This research was funded by the Natural Science Foundation of China (grant number 31500993).

## Conflict of Interest

The authors declare that the research was conducted in the absence of any commercial or financial relationships that could be construed as a potential conflict of interest.
